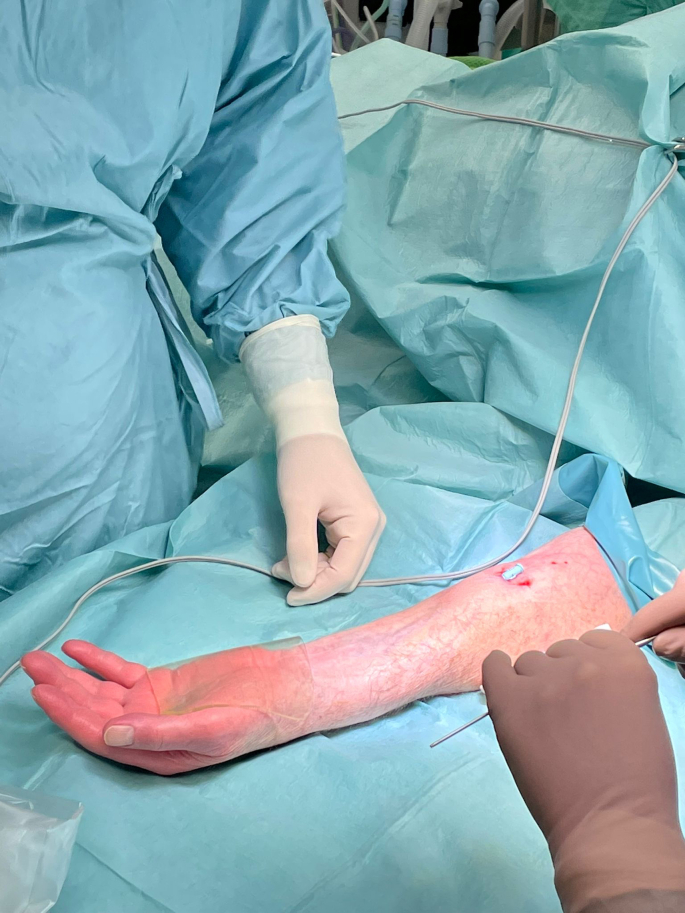# Peripheral nerve stimulation – Our experiences with the wireless high-frequency system

**DOI:** 10.1016/j.bas.2026.106112

**Published:** 2026-06-01

**Authors:** U.M. Bäzner, L. Minzenmay, C.R. Wirtz, A. Pala

**Affiliations:** Department of Neurosurgery, University of Ulm, Bezirkskrankenhaus, Günzburg

**Keywords:** Pain, Neuromodulation, Peripheral nerve surgery, Ultrasound-guided

## Abstract

**Introduction:**

Peripheral nerve stimulation using a wireless high-frequency system offers an alternative to neurostimulator implantation. This approach has the potential to reduce the treatment's risk profile and improve overall quality of life.

**Methods:**

Between January 2021 and October 2024, 21 patients were treated at our clinic using a wireless high-frequency system for peripheral nerve stimulation (PNS). The system was implanted under local anesthesia via a Tuohy needle puncture, with ultrasound guidance. The mean surgical time was 36 min (range 16 - 60 min). All patients presented with long history of chronic pain syndromes (mean 49 months, range 8-96 months) and had previously undergone multimodal pain therapy in specialized centers. The most common causes of pain were patients after traumatic peripheral nerve surgery (n = 10, 47.7%) followed by pain after previous surgeries (n = 8, 38.1%) and 3 patients (14.1%) with peripheral nerve tumors. The most common nerve targeted for stimulation was ulnar nerve (n = 7, 33.3%), followed by peroneal nerve (n = 4, 19%), tibial nerve (n = 3, 14.3%), median nerve (n = 3, 14.3%), occipital nerve (n = 2, 9.5%), infrapatellar nerve (n = 1, 4.8%) and medial antebrachii cutaneous nerve (n = 1, 4.8%). All patients reported preoperative neuropathic pain, with NRS (Numeric Rating Scale) scores ranging from 7/10 to 10/10 (mean 8.2/10).

**Results:**

Pain reduction was achieved in 17 (81%) patients, with NRS scores decreasing from 8.2/10 preoperatively to 4.3/10 postoperatively (mean reduction of 5.2/10). Additionally, 14 (66.7%) patients experienced a reduction in their pain medication use, with some patients completely discontinuing their medication.

However, no positive effects were observed in the patient receiving median nerve stimulation due to a tumor and previous fascicle biopsy, nor in the two patients with a history of lacerations and partial nerve transplants, despite correct pain area coverage.

19 (90.5%) patients would choose peripheral nerve stimulation again.

In most patients (94%, n = 16/17), stimulation was applied twice daily for 30 min. Additionally, 7/17 (41.2%) patients used the system to manage acute pain attacks. No infections, wound healing or other complications were reported in any patient.

**Conclusion:**

Peripheral wireless high frequency stimulation is a simple and feasible option for patients with chronic pain syndrome within the territory of a specific peripheral nerve. In patients with prior partial nerve grafting, seems to be the introduced system less effective, despite correct coverage of the pain area.

## Highlights

• PNS is a therapeutic option, particularly for mononeuropathies PNS should be included in the counseling of patients and expands the range of treatment options.
• Wireless systems simplify implantation and may thereby improve the risk profile and patient comfort.
• PNS should be included in the counseling of patients and expands the range of treatment options.

## 1 Introduction

Chronic neuropathic pain is a severe medical concern, often resulting from nerve injury or damage, and can be difficult to manage with traditional analgesic therapies (Bao et al., 2021). While spinal cord stimulation (SCS) and dorsal root ganglion (DRG) stimulation are well-established therapies for refractory pain, they require invasive procedures that involve the implantation of devices in close proximity to the spinal cord. These treatments are often associated with considerable risks and require ongoing maintenance (Karst, 2014). In recent years, significant advancements have been made in the field of neuromodulation, particularly in peripheral nerve stimulation (PNS) and concerning the waveforms (Pope et al., 2015; Wang and Chen, 2019). Direct nerve stimulation, has now been proven effective and safe in numerous studies (Pope et al., 2015), largely due to the reliable and complication-free placement of electrodes along nerves using ultrasound guidance. This approach has become straightforward to apply in everyday clinical practice (see Fig. 1, Fig. 2).

**Fig. 1:**
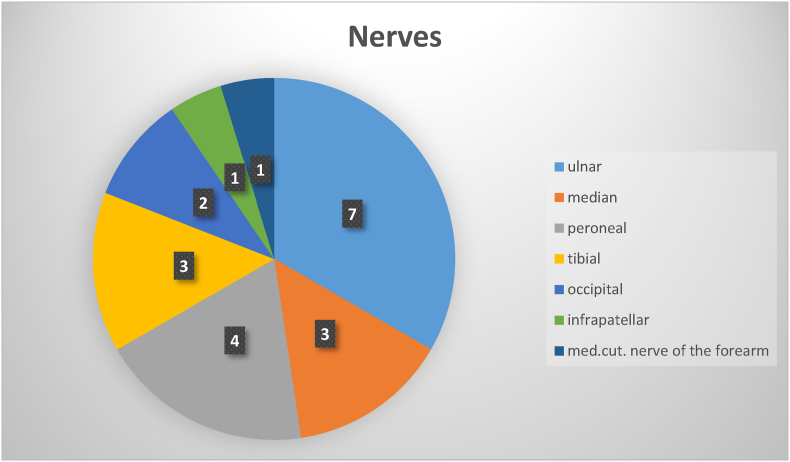
Targeted nerves for peripheral nerve stimulation (PNS).

**Fig. 2:**
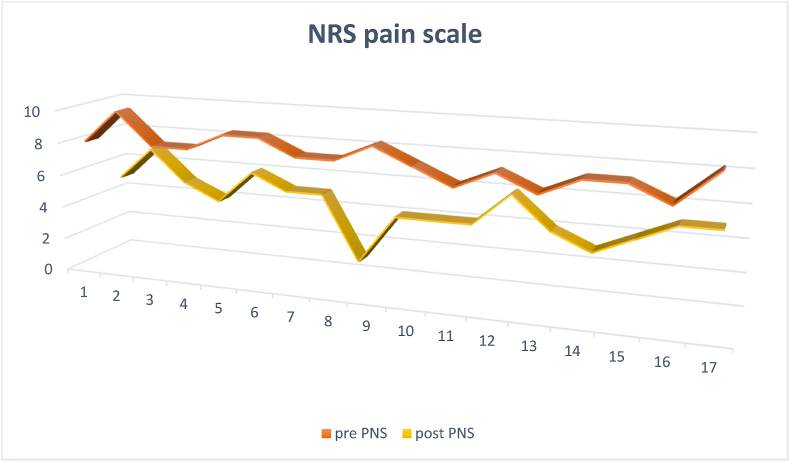
Pain reduction in 17/21 patients.

One of the major challenges in PNS has always been, and continues to be, the long distances from the nerve root to the nerves located in the extremities. These long pathways increase the risk of electrode displacement, difficulties in bridging joints, and the associated dysfunctions. As a result, many patients have historically opted for spinal cord stimulation (SCS) or dorsal root ganglion (DRG) stimulation, which are often considered more reliable for managing pain.

However, with the introduction of a new wireless system, it is possible to work without the need for extension units or implanted neurostimulators (Abd-Elsayed and Moghim, 2023). This represents a potentially valuable alternative for patients, offering a less invasive option compared to traditional methods (Eldabe et al., 2016).

In our study, we examined patients treated between 2021 and 2024 who developed chronic pain syndromes because of lesions to the peripheral nervous system. We assessed the surgical procedures, the management of the system by both the clinical team and the patients, and, most importantly, the response to stimulation. The key focus of our investigation was the reduction of pain, as well as the possibility of decreasing or eliminating the need for pain medications.

## 2 Patients and methods

So we conducted a retrospective, descriptive, observational study. A total of 21 patients (14 female, 66.7%) were treated at our clinic with the wireless high-frequency system for peripheral nerve stimulation between January 2021 and October 2024. All of these patients had presented to our specialized outpatient clinic for pain and neuromodulation and had exhausted all conservative treatment options, including multimodal pain therapy. Patients diagnosed with mononeuropathy and considered suitable candidates for PNS were selected. They were counseled about this alternative approach to SCS or DRG.

The study received approval from the local ethics committee (N 07/25) and adhered to the principles of the international Declaration of Helsinki. Informed consent was obtained from all participants.

## 3 Collected data

For each patient who received the wireless high-frequency system for peripheral nerve stimulation (PNS) during this period, a thorough preoperative assessment was conducted in our outpatient clinic. This included taking a detailed medical history, performing a neurological examination, and providing postoperative follow-up care (after 3 weeks, 3 months, 6 months, then annually or adjusted as needed). Pain levels were assessed using the Numeric Rating Scale (NRS), and medication usage was documented and adjusted accordingly.

The local medical records were analyzed for demographic data, and patients were stratified based on severity and visibility grades as proposed by Huson et al. and Ablon et al. Demographic variables such as age, sex, marital status, and employment status were also analyzed.

## 4 Statistical analysis

Descriptive statistics were used for data analysis, reporting mean and standard deviation for continuous variables and absolute and relative frequencies for qualitative parameters.

## 5 Results

This study aims to evaluate the feasibility and clinical outcome of PNS using a wireless high-frequency system in a cohort of patients suffering from chronic neuropathic pain. We explore its effectiveness, patient satisfaction, complications, and the overall impact on quality of life.

The patients were selected based on a diagnosis of chronic neuropathic pain with no satisfactory response to multimodal pain therapy. The PNS system was implanted under local anesthesia using a Tuohy needle puncture, with guidance provided by ultrasound. The entire procedure was performed in an outpatient setting, and the mean operation time was 36 min, ranging from 16 to 60 min.

All patients had a history of chronic pain, with a mean duration of 49 months (range 8-96 months). The causes of pain varied: 8 patients had undergone previous surgeries, 10 had experienced traumatic nerve injuries, and 3 had nerve tumors.

The targeted nerves for stimulation included:

Preoperatively, all patients reported neuropathic pain with an average pain score of 8.2/10 on the Numeric Rating Scale (NRS), ranging from 7/10 to 10/10.

After the implantation of the wireless high-frequency system, significant pain reduction was observed in 17 out of the 21 patients (81%). The follow-up checks are carried out after 3 weeks, 3 months, and 6 months and then annually, or adjusted as needed.

The average pain score decreased from 8.2/10 preoperatively to 4.3/10 postoperatively (mean reduction of 5.2/10). Furthermore, 14 out of the 21 patients (67%) reported a reduction in their use of pain medication, with some achieving complete cessation of their medication.

However, there were notable exceptions. One patient who underwent median nerve stimulation due to a nerve tumor and previous fascicle biopsy did not experience any positive effect. Similarly, two patients with a history of nerve lacerations and partial nerve transplants did not experience improvement, despite appropriate stimulation over the pain area.

Despite these cases, 19 out of 21 patients expressed a willingness to undergo the procedure again. One patient, however, was overwhelmed by the technical aspects of using the wireless system over several months, and two patients with negative outcomes were still generally supportive of the attempt at therapy.

The stimulation regimen typically involved two sessions per day, lasting approximately 30 min each. Seven out of 17 patients also used the system for managing acute pain attacks. No infections or wound healing problems were reported in any patient.

Thirteen out of twenty-one patients (62%) were employed prior to PNS treatment; however, due to pain, more than 50% were working reduced hours. Following stimulation, three additional patients were able to return to work, although in some cases workplace modifications were necessary.

## 6 Discussion

Peripheral nerve stimulation (PNS) using wireless high-frequency systems offers a potentially less invasive alternative, particularly in cases where the pain is localized to peripheral nerves. This technique allows for targeted nerve stimulation without the need for complex implants, offering a promising option for patients who may not be candidates for more invasive procedures. Stokey et al. (2020) were able to demonstrate this in cases of atypical facial pain. In such implantations, the use of extensions and implanted generators is often associated with dislocations and hardware-related complications.

Peripheral nerve stimulation at sites distal to the nerve root has rarely been an option due to the need for extension leads and the challenges of bridging joints. As a result, more invasive therapies such as dorsal root ganglion (DRG) stimulation or spinal cord stimulation (SCS) have often been preferred. However, many patients are critical of such a neuromodulation procedure, as they have concerns due to its proximity to the spinal cord. The direct placement of the electrode on the distal nerve under local anesthesia is better tolerated by these patients.

Our experience with wireless high-frequency peripheral nerve stimulation demonstrates that it is an effective and well-tolerated treatment option for patients suffering from chronic neuropathic pain. The system offers several advantages, including its simplicity of use, minimal invasiveness (local anesthesia, needle puncture), and relatively short procedure time. Additionally, the ability to target peripheral nerves specifically provides an opportunity to manage pain localized to particular anatomical regions, such as the ulnar, peroneal, tibial, and occipital nerves, without the need for more invasive spinal cord-based therapies (Herschkowitz and Kubias, 2018).

The clinical outcomes in our cohort are promising, with significant reductions in pain and medication use. However, there are some limitations to the effectiveness of this technique. For example, patients with prior nerve transplants or complex nerve pathologies, such as nerve tumors, may not benefit from this form of stimulation. In these cases, it appears that the underlying nerve damage may hinder the efficacy of peripheral nerve stimulation despite appropriate nerve coverage. A systematic review published in *Plastic and Reconstructive Surgery – Global Open* (Agrawal et al., 2021) discusses the use of PNS in conjunction with targeted muscle reinnervation (TMR) and regenerative peripheral nerve interfaces (RPNI). The study highlights that implanting a peripheral nerve stimulator adjacent to nerve transfers during surgery can enhance pain management and improve prosthetic use and quality of life for patients.

Additionally, Wong et al. (2022) were able to demonstrate that the effectiveness of PNS in nerve grafts is believed to stem from its ability to activate Aβ fibers, which can modulate pain signals through the dorsal root ganglion, thereby blocking chronic pain impulses transmitted via C fibers. Additionally, PNS may influence neuroinflammatory pathways and enhance descending pain inhibition, contributing to pain relief.

This stands in contrast to the results of our study, and the techniques used also differ. Certainly, the sample sizes remain too small, and the cases overall are too heterogeneous.

Importantly, there were no reports of complications such as infection or poor wound healing, which are commonly associated with more invasive procedures. This highlights the safety of the wireless high-frequency system, particularly in terms of its minimal invasiveness.

While the wireless system appears to be a feasible and effective option for managing localized chronic pain, the long-term benefits of this approach need to be further evaluated. Patients must also be educated on the technical aspects of the system to ensure optimal outcomes, as demonstrated by one patient’s challenges in adapting to the technology.

One patient of our study felt overwhelmed by the technical aspects of using the system over time, but even patients with negative outcomes generally supported the attempt at therapy.

Most published studies focus primarily on peripheral nerve field stimulation (Deogaonkar and Slavin, 2014). Our study specifically addresses the stimulation of a single, individual peripheral nerve in patients with consequent pain syndromes. There is still a lack of sufficient studies on this topic, with most observations being case reports or studies focusing on specific pain syndromes.

In this context, Bayerl et al. (Früh et al., 2023) demonstrated that the use of this system, which we also employ, for infrapatellar nerve stimulation in patients with chronic knee pain leads to a significant improvement in the short-term course.

Regarding occipital neuralgia, Add-Elsayed et al. (2024) reported improvements in pain relief in three cases using this system (Abd-Elsyed et al.). They suggested it as an alternative treatment after previous multimodal pain therapy and compared it to systems involving implanted neurostimulators.

Additionally, Manzi et al. (Manzi et al.) demonstrated an improvement in quality of life and pain relief in patients with chronic shoulder pain through stimulation of the suprascapular nerve.

The key difference between our study and others is that we did not, for example, select patients based on shoulder pain or knee pain as the primary criterion—focusing on the symptom itself. Instead, we chose patients who had specific lesions, trauma, or prior surgeries on peripheral nerves, such as the ulnar nerve (e.g., after multiple decompression surgeries). Our approach is different in that we targeted patients based on their peripheral nerve conditions rather than the general symptom of pain.

### 6.1 Limitations

Limitations of this study certainly include the small patient cohort and the heterogenity of the stimulated nerves as well as the overall variety of clinical conditions. However, the primary aim of this study is to introduce this new system, and it is important first to evaluate these initial cases in order to enable more precise patient selection and to improve implantation procedures.

As with any form of neuromodulation, a placebo effect is likely present initially, making long-term follow-up essential to better assess the durability of the effects. Pain is fundamentally a subjective symptom, and measurement methods and pain scales are susceptible to bias and individual variability.

In future studies with a larger cohort, we plan to include additional questionnaires addressing quality of life (QoL), depression and sleep.

## 7 Conclusion

Wireless high-frequency peripheral nerve stimulation represents a promising, less invasive alternative to more traditional neurostimulation therapies such as SCS or DRG stimulation. This approach is particularly valuable for patients with peripheral nerve pain localized away from the nerve root. The procedure is simple, quick, and well-tolerated, offering significant pain relief and reduced medication use in most patients. However, its effectiveness may be limited in cases involving complex nerve pathologies, such as nerve transplants or tumors. Nonetheless, this technique holds promise for expanding the treatment options available to patients with chronic neuropathic pain and may be an important tool in the management of such conditions. Further studies are needed to confirm these findings and assess long-term outcomes.

## Declaration of competing interest

The authors declare that they have no known competing financial interests or personal relationships that could have appeared to influence the work reported in this paper.

## Figures and Tables

**figs1:**
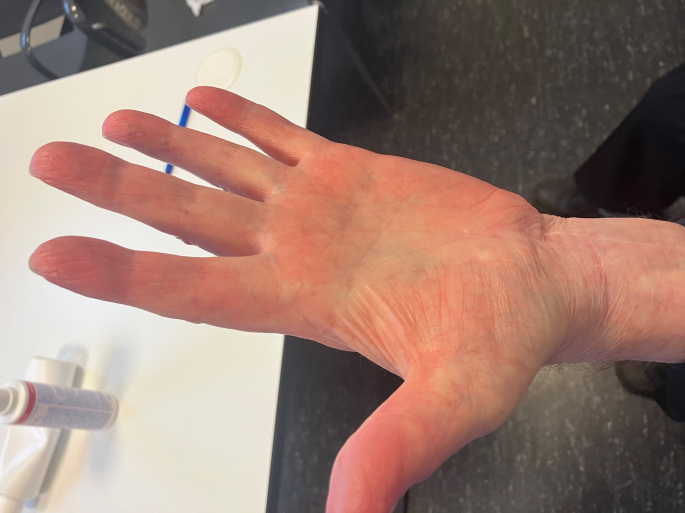


**figs2:**